# Does Absorbable Mesh Prevent Recurrent Ptosis after Mastopexy?

**DOI:** 10.1007/s00266-022-03124-x

**Published:** 2022-10-06

**Authors:** Eric Swanson

**Affiliations:** Swanson Center, 11413 Ash St, Leawood, KS 66211 USA

*Level of Evidence V* This journal requires that authors assign a level of evidence to each article. For a full description of these Evidence-Based Medicine ratings, please refer to the Table of Contents or the online Instructions to Authors www.springer.com/00266.

Inclusion of mesh at the time of mammaplasty is a recurring theme at plastic surgery meetings and in publications. Mallucci and Bistoni [1] conclude that GalaFLEX poly-4-hydroxybutyrate (P4HB) mesh (BD, Franklin Lakes, N.J.) provides long-term stability of the lower pole after mastopexy and augmentation/mastopexy. Although two authors are listed, the singular “author” is used throughout the article, and the authors report a single surgeon experience. Mallucci and Bistoni [1] report full correction of ptosis in all study groups (mastopexy and augmentation/mastopexy, primary and secondary), consistent maintenance of lower pole position, and no recurrence of ptosis or need for reoperation. Dr. Mallucci routinely inserts this product in all mastopexies and in small to moderate breast reductions [1].

**Fig. 1:**
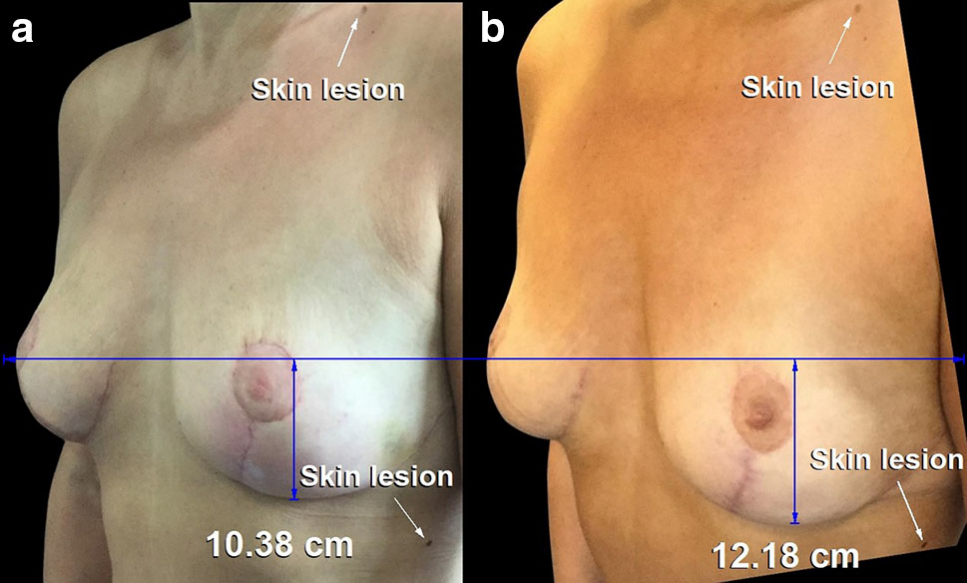
**a**, **b** This 52-year-old woman is shown 3 months and 1 year after a mastopexy including P4HB mesh. The authors report that the photographs show stability. The images have been matched for size and orientation using the Canfield 7.4.1 Mirror imaging software (Canfield Scientific, Fairfield, N.J.), correcting a 9° upward tilt of the later photograph compared to the earlier photograph, and accounting for the oblique margins. To match the photographs, two conspicuous pigmented skin lesions were labeled. **a**: although the earlier photograph was reportedly taken 3 months after surgery, redness and swelling are still visible. **b**: The left lower pole level has descended 1.8 cm. A 32.5 cm upper arm length was used for calibration. Adapted from Figure 13, Mallucci and Bistoni [1]

**Fig. 2:**
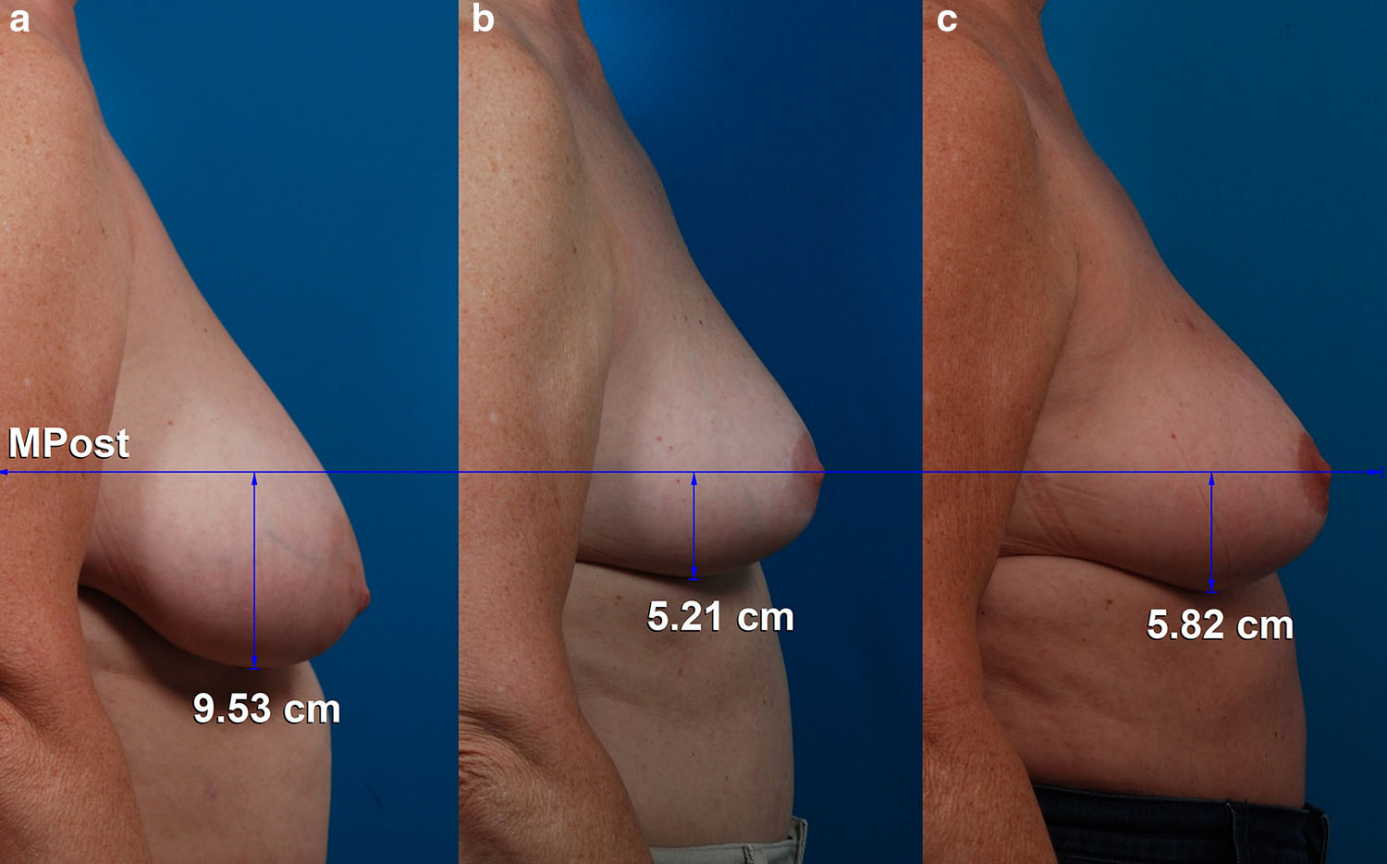
**a**, **b**, **c** This 55-year-old woman is shown before, 1 year, and 10 years after a vertical breast reduction using a medial pedicle. The right resection weight was 360 g. She had a simultaneous abdominoplasty and liposuction of the abdomen and flanks. **b**, **c** The lower pole level has descended 0.6 cm between 1 and 10 years after surgery. The nipple level is unchanged. *MPost*, plane of maximum postoperative breast projection

Commentary is needed regarding the study method. Although the title indicates 100 cases, measurements were performed on only 65 patients [1]. The median follow-up time was 14 months, which is too short to assess long-term changes. There was no control group; all patients received mesh. Highly cropped, nonstandardized, oblique photographs were evaluated, which are not generally used for measurements because of difficulty exactly matching the degree of rotation (Fig. 1) [2]. Standardized lateral photographs are preferred because they allow comparison of the gland level and nipple level (Fig. 2) [2].

The authors used photograph editing software for measurements, not a tape measure. Without photographic calibration, only percent changes were possible. Measurements were made from the nipple to the inframammary fold (IMF), both vertically and along the curvilinear surface [1]. It is unclear how such measurements were possible, with the IMF tucked in the fold under the breast and not visible in photographs depicting breast ptosis. (This is a limitation when using the IMF as a landmark and one that is avoided by using the lower pole level as a landmark instead.) [2] No measurements are included on any of the photographs [1]. The authors list percent changes but do not indicate right or left sides, or whether these percentages represent an average of the two sides. The authors tabulate the descent of the IMF based on the nipple-IMF measurements, which presumes that the nipple position is static. The photographs differ in magnification, tilt, and rotation (Fig. 1), confounding measurements. Without matched standardized photographs, any attempts to measure dimensions, particularly when one landmark (the IMF) is hidden, are unreliable [2].

When the images are matched, to the extent that this is possible, using static pigmented skin lesions, descent of the lower pole and nipple is evident (Fig. 1), despite inclusion of mesh. In many of the authors’ postoperative photographs, the upper pole appears deflated and the nipple rotates upward, giving the appearance of pseudoptosis [1]. The inframammary scars are long, sometimes extending laterally past the anterior axillary line and connecting across the sternum [1]. In the patient depicted in the authors’ figure 9 [1], the photograph is reversed postoperatively so that the right breast now appears to be the left breast, as evidenced by the mole patterns.

The authors reference measurement studies by Swanson but state that changes in breast dimensions over time are less well-documented [1]. On the contrary, a 2018 measurement study evaluated patients at 10 years’ follow-up [2]. This long-term study showed that breast shape holds up well when using the vertical mammaplasty technique. There was no significant change in nipple level between 1 and 10 years in women treated with vertical mastopexy. In patients treated with vertical mastopexy and augmentation/mastopexy without mesh, the lower pole descended only about 1 cm, on average (Fig. 2) [2].

A vertical mammaplasty, uniquely, elevates the IMF [2]. The distance from the areola to the IMF (the lower pole arc) is reduced postoperatively in women treated with a vertical breast reduction [3]. Elongation of the lower pole occurs after a Wise pattern inferior pedicle breast reduction, even when limiting the vertical limb to 5 cm [2]. Mallucci and Bistoni [1] report lengthening of the lower pole arc, despite the addition of mesh.

These findings point to an advantage of the vertical mammaplasty over techniques that preserve a lower pole parenchymal flap. Using the vertical technique, a wedge is removed from the lower pole and the pillars are cinched together, providing maximum lift, and converting width to projection, a favorable trade [2]. The Wise pattern does the reverse, trading projection for width. The authors maintain the nipple and areola on a superior pedicle. The mesh is applied to the lower pole parenchyma, which is preserved [1]. Placing a layer of mesh over this bulge does not appear to prevent its descent (Fig. 1). Parenchymal resection avoids a persistent lower pole bulge that may require secondary correction [2]. To use this overlay method, a suboptimal parenchymal dissection is needed — one that preserves tissue in the exact location where it needs to be removed. Additional skin flap undermining is necessary, increasing the risk of ischemic complications [3].

Proponents of mesh often use the terms hammock, scaffold, or sling, suggesting that the mesh holds the tissue up like a sling [1, 3]. However, claims of a reduced risk of recurrent ptosis have not been substantiated with measurements [3]. A recent systematic review found that implanted mesh does not prevent recurrent ptosis and bottoming out after mastopexy [3]. Problems with studies include poor outcome measures, inherent bias, and a low level of evidence [3]. Photographs are compromised by lack of standardization, and differing magnification, orientation, and tilt (Fig. 1) [2]. Institutional review board approval is often neglected [1], but is not optional. Off-label regulatory status of this product [3] requires disclosure.

Importantly, patient-reported outcome data are frequently missing from studies of mesh [1]. The authors dismiss the feasibility of a control group. This could be done using a historical case–control study design, provided photographs are available of earlier mastopexy patients treated without mesh. Proper photographic standardization is essential [2]. The authors do not mention cost. The material remains palpable for about 1 year and may interfere with mammography [3]. Importantly, neither mesh nor acellular dermal matrix is approved by the U.S. Food and Drug Administration for breast surgery [3].

Conflict of interest is a major consideration when evaluating studies of mesh. In their disclosure [1], the authors disclose only that Dr. Mallucci is a minor shareholder for Polytech (Dieburg, Germany), “unrelated to the use of P4HB in any way.” However, in a simultaneous publication [4], co-authored by Dr. Mallucci, the disclosure paragraph reveals that he is also a consultant for BD. BD is the company that acquired Tepha Inc. in July 2021, and the manufacturer of P4HB mesh. The authors do not mention whether they received the product at a discounted price.

Patients and plastic surgeons who might be tempted to use this product off-label should be informed that there is no controlled study with measurements that support claims of improved long-term stability. The only published systematic review finds no benefit [3]. Until it is clear that mesh provides a proven advantage, and one that justifies its cost and a suboptimal dissection for placement, it is overstepping to recommend its routine incorporation in all cases of mastopexy and augmentation/mastopexy.
